# Island biogeography and human practices drive ecological connectivity in mosquito species richness in the Lakshadweep Archipelago

**DOI:** 10.1038/s41598-022-11898-y

**Published:** 2022-05-16

**Authors:** Muhammad Nihad P. P., Rohini P. D., Sutharsan G., Anagha Ajith P. K., Sumitha M. K., Shanmuga Priya A., Rahul P., Sasikumar V., Shaibal Dasgupta, Jayalakshmi Krishnan, Farah Ishtiaq

**Affiliations:** 1 Tata Institute for Genetics and Society, New InStem Building, GKVK Post, Bellary Road, Bangalore, 560065 India; 2 Department of Life Sciences, Central University of Tamil Nadu, Thiruvarur, Tamil Nadu 610005 India

**Keywords:** Ecology, Environmental sciences, Diseases

## Abstract

Mosquitoes are globally distributed and adapted to a broad range of environmental conditions. As obligatory hosts of many infectious pathogens, mosquito abundance and distribution are primarily determined by the presence and quality of larval habitats. To understand the dynamics and productivity of larval habitats in changing island environments, we conducted a four-month mosquito survey across ten inhabited islands in the Lakshadweep archipelago. Using fine-resolution larval habitat mapping, we recorded 7890 mosquitoes representing 13 species and 7 genera. Of these, four species comprised 95% of the total collections—*Aedes albopictus* (*Stegomyia*) was the dominant species followed by *Armigeres subalbatus*, *Culex quinquefasciatus* and *Malaya genurostris*. We found larval species richness was positively associated with the island area and mosquito larval richness (Chao1 estimator) was higher in artificial habitats than in natural habitats. Furthermore, mosquito species composition did not deteriorate with distance between islands. Mosquito abundance by species was associated with microclimatic variables—pH and temperature. We detected co-existence of multiple species at a micro-habitat level with no evidence of interactions like competition or predation. Our study analyzed and identified the most productive larval habitats –discarded plastic container and plastic drums contributing to high larval indices predicting dengue epidemic across the Lakshadweep islands. Our data highlight the need to devise vector control strategies by removal of human-induced plastic pollution (household waste) which is a critical driver of disease risk.

## Introduction

The central goal in ecology is to understand how species colonization, extinction and adaptation to new habitats shape the patterns in diversity and richness of ecosystems (e.g.,^1,2^). Mosquitoes (Diptera: Culicidae) are classified as the world’s deadliest animals responsible for deaths of more than one million people every year^3^. With increase in the incidence of mosquito-borne diseases and range expansion of primary mosquito vector species to new habitats, it is important to understand the drivers of species diversity and richness from both ecological and epidemiological perspectives. One of the most important determinants of mosquito colonization is the presence and quality of larval habitat^4^. Larval habitats are closely associated with female oviposition preferences^5^. Both biotic attributes such as conspecific larval density (i.e., competition) and diversity of host (i.e., presence of predators), nutritional resources (e.g., organic matter) and abiotic properties (pH, temperature, salinity etc.) of the aquatic habitat determine the fitness (survival, growth rate and body size) of surviving larvae^6^. Changing land-use patterns, urbanization and environmental conditions (temperature, humidity, availability of larval habitat) can have dramatic effects on their survival, distribution and density^7^. Therefore, to assess and predict the disease risk with changing practices and to quantify the impact of human activities, it is important to assess and understand how mosquito larval ecology and population dynamics are related to fluctuations in their environments. Urbanization and climate are driving genetic and ecological changes (e.g., dry season intensity and human population density) in mosquito species with narrower ecological niches, causing a behavioural shift towards human-biting^8^. For example, to transition from ancestral forest habitat to human settlements, *Aedes aegypti* developed a preference for human blood and use of man-made containers as larval habitats^9–11^.

Islands as natural laboratories are important model systems for studying fundamental questions related to disease ecology, evolutionary biology, biogeography, and epidemiology^1,12,13^ related to the origin of vector populations and disease control^14^. As isolated geographical entities, islands provide ideal ecosystems to tease apart patterns in colonization, extinction, distribution and dynamics of mosquito species in relation to human activities.. Islands have also played an instrumental role in the development of several fundamental theories including one of the most robust generalizations in ecology – the species–area relationship (SAR) which predicts that the number of species is a function of area^15,16^. Whilst a positive correlation between species numbers with area has been recognized among organisms (see^17^), it was further promoted by MacArthur and Wilson^1^ through the equilibrium theory of island biogeography (ETIB).

The ETIB predicts that species richness increases with area which represents a dynamic equilibrium between immigration and extinction rates, which is affected by the size of the island and the distance to the source of colonization^1^. Therefore, species-area relationships are fundamental to understanding patterns of species diversity and richness and for predicting species extinction risk in response to climate change^18^ as well as colonization rates.

The SAR is commonly described by the power model, which in its logarithmic form is given by log*S* = logC + *z*log*A* (where *S* = island species richness, *A* = island area, and *z* and logC are fitted parameters representing the slope and intercept of the model, respectively). In the context of mosquito, extinction risks of a species can be related to species traits such as mobility, diversity of larval habitats, tolerance to saline habitat etc^19^. A combination of these traits allows a mosquito species to colonize or replace other mosquito species as more suitable habitat and conditions facilitate the process of natural colonization^20^. Given the environmental conditions are favorable, it takes several generations for a mosquito species to successfully colonize a new location to the levels of abundance that allow for detection during active surveillance. For example, an unseasonal outbreak of malaria reported in Djibouti city in the Horn of Africa was caused by recent invasion of urban Asian malaria mosquito *Anopheles stephensi*^21^. In addition, many species remain undetected despite active surveillance, for example by using genetic analyses of *Aedes aegypti*, it was revealed that the California population had been around at least 30 years prior to its discovery in 2013^22^.

Finally, decay in species composition with increasing distance between island populations (beta diversity or species turnover^23^) is a universal biogeographic pattern observed across communities^24–26^. Therefore, understanding underlying causes of distance-decay patterns can provide insights into the mechanism maintaining biodiversity. Beta diversity can depend on various processes—it can be reduced by strong competitive exclusion or dispersal limitation or high rates of random extinction and immigration events leading to demographic stochasticity^27,28^ and heterogeneous distribution (ecological drift) on short time scales or genetic processes allowing for taxon diversification on longer time scales^23^. For mosquito communities, beta diversity and distance-decay patterns in community similarity could be driven by ecological connectivity, environmental conditions, or heterogeneity in habitat^29^ larval density is dependent of microclimate (temperature and pH)^30,31^ and habitat availability^32^.

The archipelago of Lakshadweep [10.57° N and 72.64° E] comprises of 36 islands in the Arabian sea and is scattered over approximately 75,000 sq km and nearly 200–400 km off the south-western coast of India. Kavaratti and Minicoy are the largest islands. The climate is tropical, humid, and warm. The Lakshadweep archipelago has served as a maritime stopover for centuries^33^. With both entomological and epidemiological perspectives, sporadic surveys have been conducted on these islands. For example, *Aedes aegypti* was detected in Minicoy in 1974^34^. In 2000, *Aedes aegypti* larvae were collected from Kavaratti, Agatti, and chikungunya antibodies along with *Aedes* sp. were reported from Kadmat, Amini and Kavaratti islands^35^. However, no study has ever explored with the effects of accessibility to the mainland source, vector diversity or population densities to estimate the related disease risks. In 1958, filariasis was reported as endemic to these islands ^36^. Subsequently, sporadic cases of malaria were reported in 1978^37^. However, the presence of *An. stephensi*, a vector of urban malaria was not reported until 2000 from two islands—Agatti and Kavaratti^34^. Similarly, in 2009 *Aedes albopictus*, a globally important vector of dengue and chikungunya virus was reported as the predominant species on the Lakshadweep islands^38,39^. Based on a recent mosquito survey in the Lakshadweep islands, *Aedes* sp. entomological indices were reported above the epidemic threshold as defined by the World Health Organisation (WHO), indicating a high risk for dengue virus transmission^40^. Due to its geographic location and ecological connectivity with the mainland, the mosquito fauna of Lakshadweep offers unique insights into the process of colonization and extinction using an island biogeographic approach and to understand the impact of urbanization on this important taxonomic assemblage.

In this study, we used larval and adult mosquito field surveys to understand whether mosquito species richness and composition in oceanic islands are predicted by the theory of island biogeography. We wanted to understand how larval habitat creates a diverse array of microclimate (temperature and pH) which can further influence species diversity and distribution on an island. Defining how mosquito vector species composition and abundance depend on environmental resources across habitats can be helpful in identifying and implementing different types of vector control strategies. Therefore, our goal was to analyze and identify the most productive larval habitats and consequently, potentially increased risk of disease transmission on Lakshadweep islands. Specifically, our main aims were to understand:

i. What are the patterns in mosquito species diversity and distribution near human dominated areas on these islands?
ii. Does the mosquito species richness follow a species-area relationship?
iii. How mosquito beta diversity varies over spatial scale?
iv. Is larval abundance associated with habitat type or microclimate?
v. How does mosquito assemblages exist in different habitats?
vi. How does dengue epidemic threshold vary by island based on *Aedes* larval indices?

## Results

### Mosquito species diversity and richness

A total of 7890 mosquitoes comprising 13 species from 7 genera were collected in ten islands from October 2019 to January 2020 using larval and adult collection methods. Among these, *Aedes albopictus* was the dominant species 4617 (59%), followed by *Armigeres subalbatus* 2159 (27%), *Culex quinquefasciatus* 572 (7%) and *Malaya genurostris* 153 (2%). These four species comprised 95% of total collected specimens.

Using larval sampling, a total of 3031 potential larval habitats were surveyed, and 6017 mosquitoes were collected. Of these 4378 were *Aedes albopictus* (74%), followed by *Armigeres subalbatus* 910 (15%), *Malaya genurostris* 153 (2.5%) and *Culex quinquefasciatus* 152 (2.5%), comprising of 94% of all collected mosquitoes. The most productive aquatic habitats supporting high larval abundance of *Aedes albopictus* in the Lakshadweep islands were discarded plastic containers (DP), plastic drums (PD), buckets (BU), tyres (TY) representing approximately 89% of all *Aedes albopictus* collected (Fig. S1).

**Figure 1:**
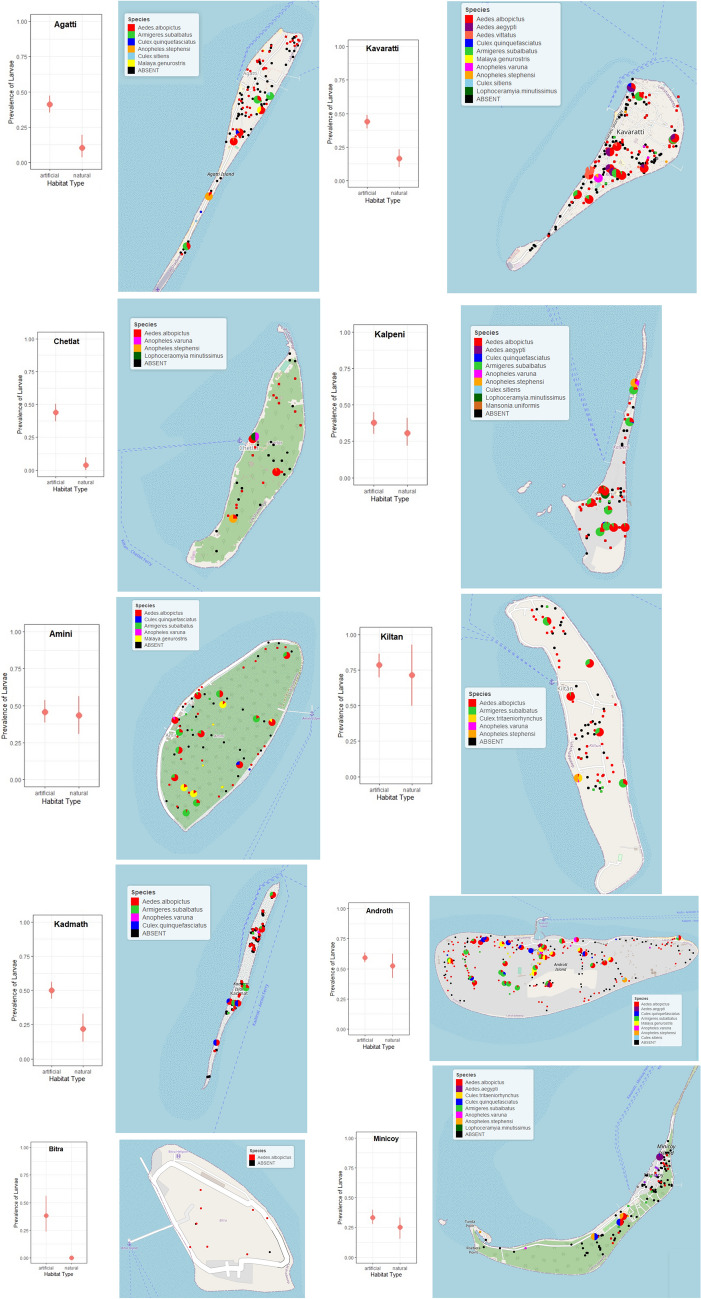
Mosquito larval survey sites shown in coloured dots on ten Lakshadweep Islands. The mean and 95% confidence interval bar show prevalence of artificial and natural habitats on each island. Maps produced using *leaflet* package in R.

Using adult collection methods, the sampling effort varied on each island (Table S1). A total 1873 mosquitoes were collected. Of these 1249 were *Armigeres subalbatus* (67%), 420 *Culex quinquefasciatus* (22%) and *Ae. albopictus* 184 (10%), comprising 99% of total collected specimens. There was a high possibility of underestimating species richness using adult sampling methods—*Lutzia fuscana* was missing in larval collections and *Anopheles varuna*, *Malaya genurostris, Mansonia uniformis* and *Culex tritaeniorhynchus* in adult collections (Table S2) (Fig. 1).

**Figure 2:**
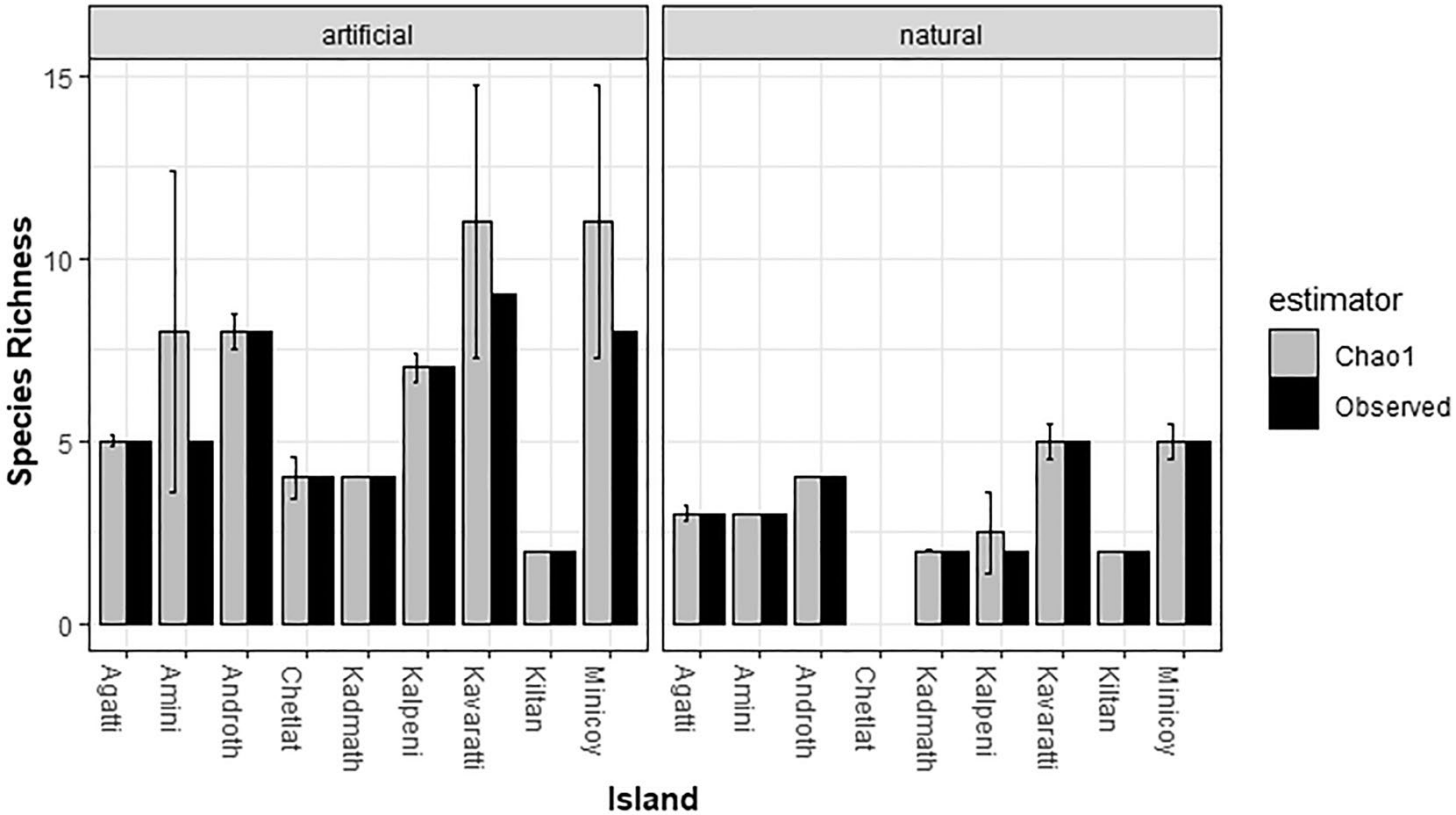
Observed and Chao 1 estimated mosquito richness in natural and artificial larval habitats on each island. The bars represent standard error.

In general, mosquito larval richness (Chao1 estimator) was higher in artificial habitats than in natural habitats (Fig. 2). Our individual-based rarefaction curves indicated that sampling of the mosquito species reached an asymptote in natural habitat, whereas mosquito diversity remained under-sampled in Kavaratti, Minicoy and Amini in artificial habitats. Similarly, the rarefaction analysis based on adult sampling showed that Bitra and Kavaratti remained under-sampled (Fig. S2).

**Figure 3:**
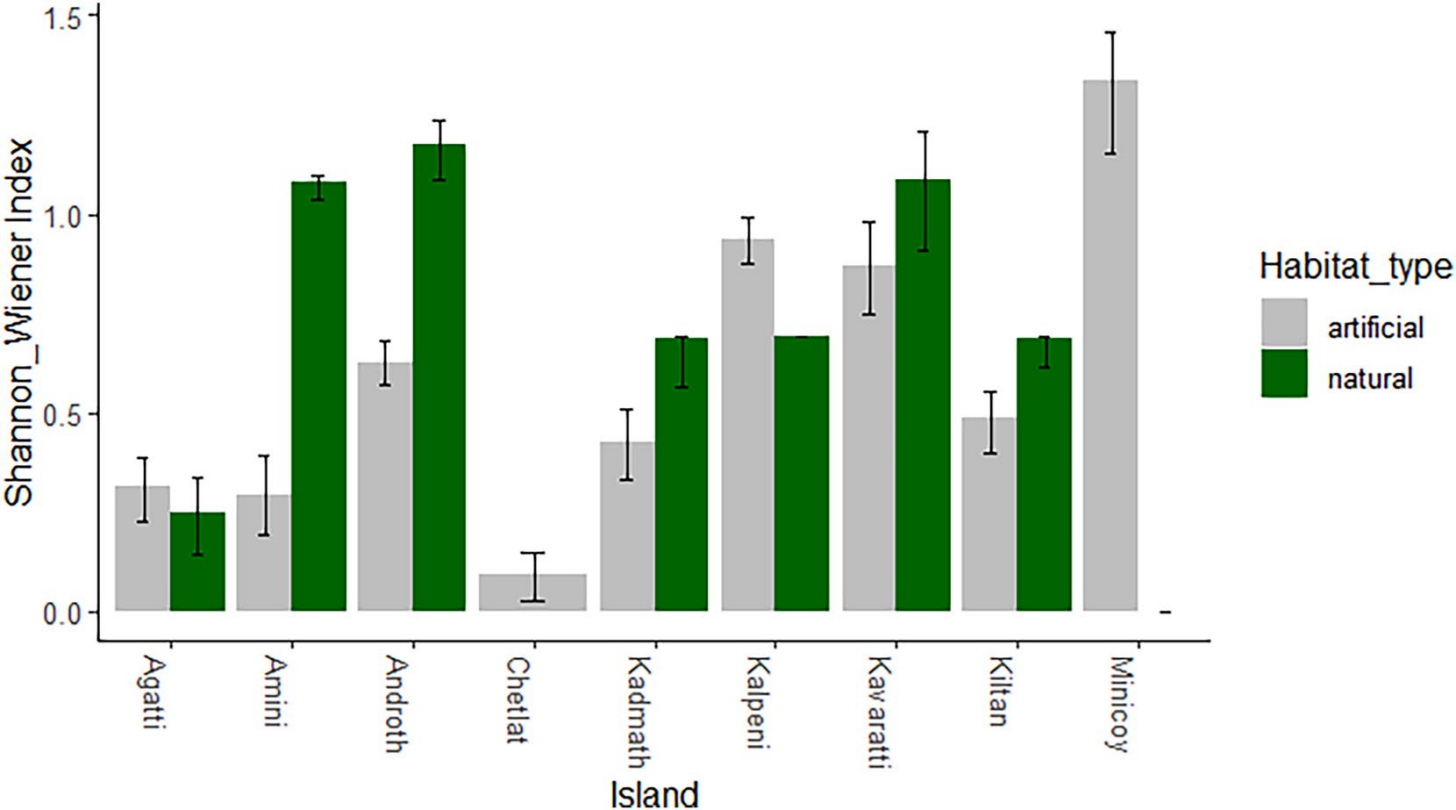
Larval diversity in natural and artificial habitats as estimated by Shannon Wiener diversity index.

Alpha diversity analysis revealed variations in mosquito diversity among collection methods. The larval collection method captured significantly higher diversity than adult collection methods (Kruskal–Wallis chi-squared = 4.37, df = 1, p < 0.03). Using the Shannon–Wiener index, larval mosquito diversity varied with habitat type (natural versus artificial) across islands – natural habitats exhibited higher indices than artificial habitats except Kalpeni, Chetlat and Minicoy (Fig. 3).

### Species-area relationship

**Figure 4:**
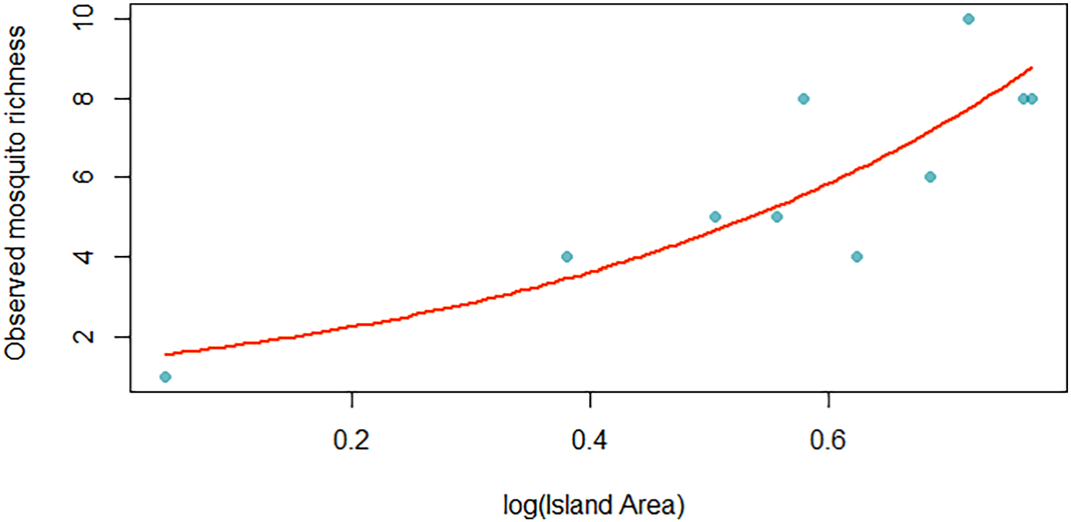
Species-area relationship between log area and mosquito richness found in the Lakshadweep Islands.

We found larval species richness was positively associated with the island area (*S* = 0.33 + 2.38 log *AREA* where 0.33 is the fitted intercept and 2.38 is the fitted slope), the best model predicting the species-area relationship (ΔAICc = 0.00, Fig. 4). This model provided the highest explanatory power (pseudo-R^2^= 0.74), and it was statistically significant (*p* value = 0.007) of all the models analyzed (Table 1). We did not find any significant relationship in adult species richness with any of these models.

**Table 1:** Candidate models for predicting mosquito species richness in the Lakshadweep islands.

Larval richness models	Intercept	Slope	AIC	AICc	Weight
Model set1					
*S* = a + b*log *Area*	**0.33 (0.57)**	**2.38 (0.88)**	**43.9**	**0.00**	**0.916**
*Null model*	1.77 (.013)		49.9	5.99	0.046
S = a + b*log *Proximity*	0.78 (0.67)	0.57 (0.37)	50.7	6.77	0.031
S = a + b*log *Area* + c*log *Proximity* + d*log Area* log *Proximity*	− 2.90 (5.79)	*b* = − 6.88 *c* = 1.94 *d* = − 2.65	53.6	9.71	0.007
Model set 2					
*S* = a + b*log *Area*	**0.33 (0.57)**	**2.38 (0.88)**	**43.9**	**0.00**	**0.932**
*Null Model*	1.77 (0.13)		49.9	5.99	0.047
*S* = a + b*log *Isolation (Calicut)*	4.85	− 1.21	52.5	8.62	0.012
*S* = a + b*log *Area* + c*log *Isolation* + d*log *Area* * log *Isolation* *(Calicut)*	26.87	*b* = − 32.39 *c* = − 10.44 *d* = 13.44	53.1	9.22	0.009
Model set 3					
*S* = a + b*log *Area*	**0.33 (0.57)**	**2.38 (0.88)**	**43.9**	**0.00**	**0.932**
*Null Model*	1.77		49.9	5.99	0.047
*S* = a + b*log *Isolation (Mangalore)*	− 2.04	1.52	51.8	7.87	0.018
*S* = a + b*log *Area* + c*log *Isolation* + d*log *Area* * log *Isolation* *(Mangalore)*	− 2.04	*b* = − 0.12 *c* = − 0.68 *d* = 1.00	54.2	10.28	0.005
Model set 4					
*S* = a + b*log *Area*	**0.33 (0.57)**	**2.38 (0.88)**	**43.9**	**0.00**	**0.892**
*S* = a + b*log *Isolation (Kochi)*	10.48	− 3.36	49.6	5.63	0.053
*Null Model*	1.77		49.9	5.99	0.045
*S* = a + b*log *Area* + c*log *Isolation* + d*log *Area* * log *Isolation* *(Kochi)*	24.08	*b* = 28.31 *c* = − 9.01 *d* = 11.62	53.0	9.06	0.010
Adult richness Models	Intercept	Slope	AIC	AICc	Weight
Model set 1					
*S* = a + b*log *Area*	1.10	1.31	46.2	0.00	0.45
*Null Model*	1.87		46.8	0.56	0.34
S = a + b*log *Proximity*	0.97	0.52	47.8	1.58	0.20
S = a + b*log *Area* + c*log *Proximity* + d*log Area* log *Proximity*	− 1.99	*b* = 5.24 *c* = 1.85 *d* = -2.33	55.4	9.16	0.005
Model set 2					
*S* = a + b*log *Area*	1.10	1.31	46.2	0.00	0.45
*Null Model*	1.87		46.8	0.56	0.34
*S* = a + b*log *Isolation (Calicut)*	3.86	− 0.78	49.7	3.51	0.08
*S* = a + b*log *Area* + c*log *Isolation* + d*log *Area* * log *Isolation* *(Calicut)*	10.02	*b* = − 10.08 *c* = − 3.44 *d* = 4.43	56.3	10.10	0.03
Model set 3					
*S* = a + b*log *Area*	1.10	1.31	46.2	0.00	0.506
*Null Model*	1.87		46.8	0.56	0.383
*S* = a + b*log *Isolation (Mangalore)*	− 0.75	1.04	49.3	3.10	0.108
*S* = a + b*log *Area* + c*log *Isolation* + d*log *Area* * log *Isolation* *(Mangalore)*	4.50	*b* = − 4.18 *c* = − 1.36 *d* = 2.19	56.5	10.23	0.003
Model set 4					
*S* = a + b*log *Area*	1.10	1.31	46.2	0.00	0.470
*Null Model*	1.87		46.8	0.56	0.355
*S* = a + b*log *Isolation (Kochi)*	7.81	− 2.29	48.2	2.01	0.172
*S* = a + b*log *Area* + c*log *Isolation* + d*log *Area* * log *Isolation* *(Kochi)*	8.72	*b* = − 6.32 *c* = − 2.87 *d* = 2.83	56.2	9.95	0.003

### Distance-decay relationship

**Figure 5:**
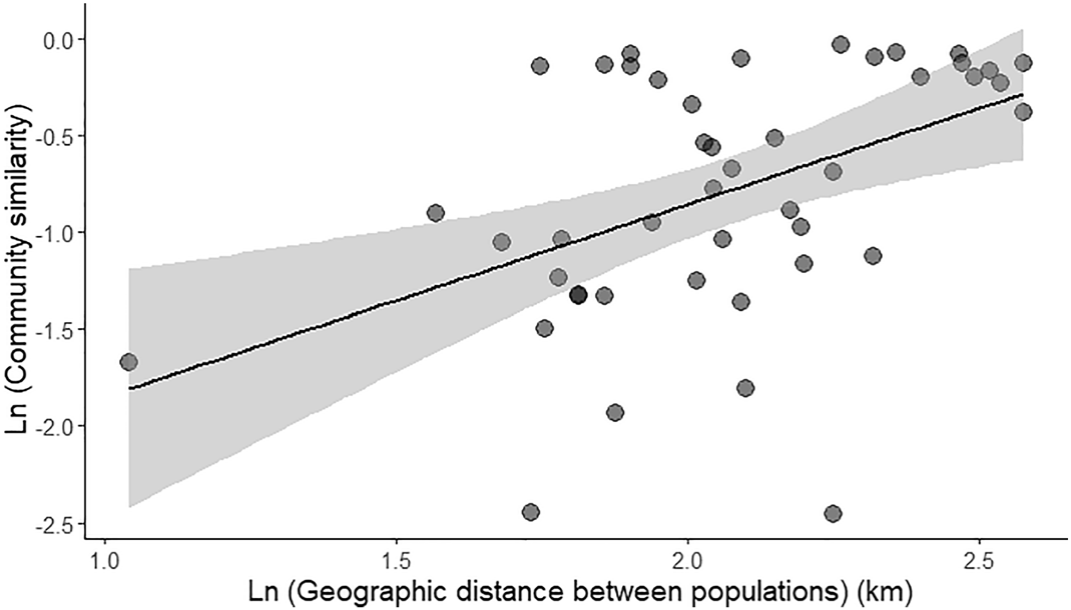
Distance-decay relationship between mosquito species across the Lakshadweep islands.

A plot of community similarity versus geographic distance for each pairwise islands revealed that the mosquito community display a significant positive distance-decay curve (slope = 0.30, Mantel statistic *r* = 0.46, *p* < 0.03). This further suggest that mosquito species composition do not deteriorate with increase in distance between islands (Fig. 5).

### Association between larval abundance and habitat types

We found artificial habitats were significantly more prevalent than natural habitats on Agatti (χ^2^ = 22.18, *p* < 0.001), Chetlat (χ^2^= 37.65, *p* < 0.001), Kadmath (χ^2^ = 17.26, *p* < 0.001) and Kavaratti (χ^2^ = 33.73, *p* < 0.001; Fig. 1). Furthermore, the mosquito abundance was lower in natural habitats than artificial habitats (β =  − 0.71, *p* < 0.02).

**Figure 6:**
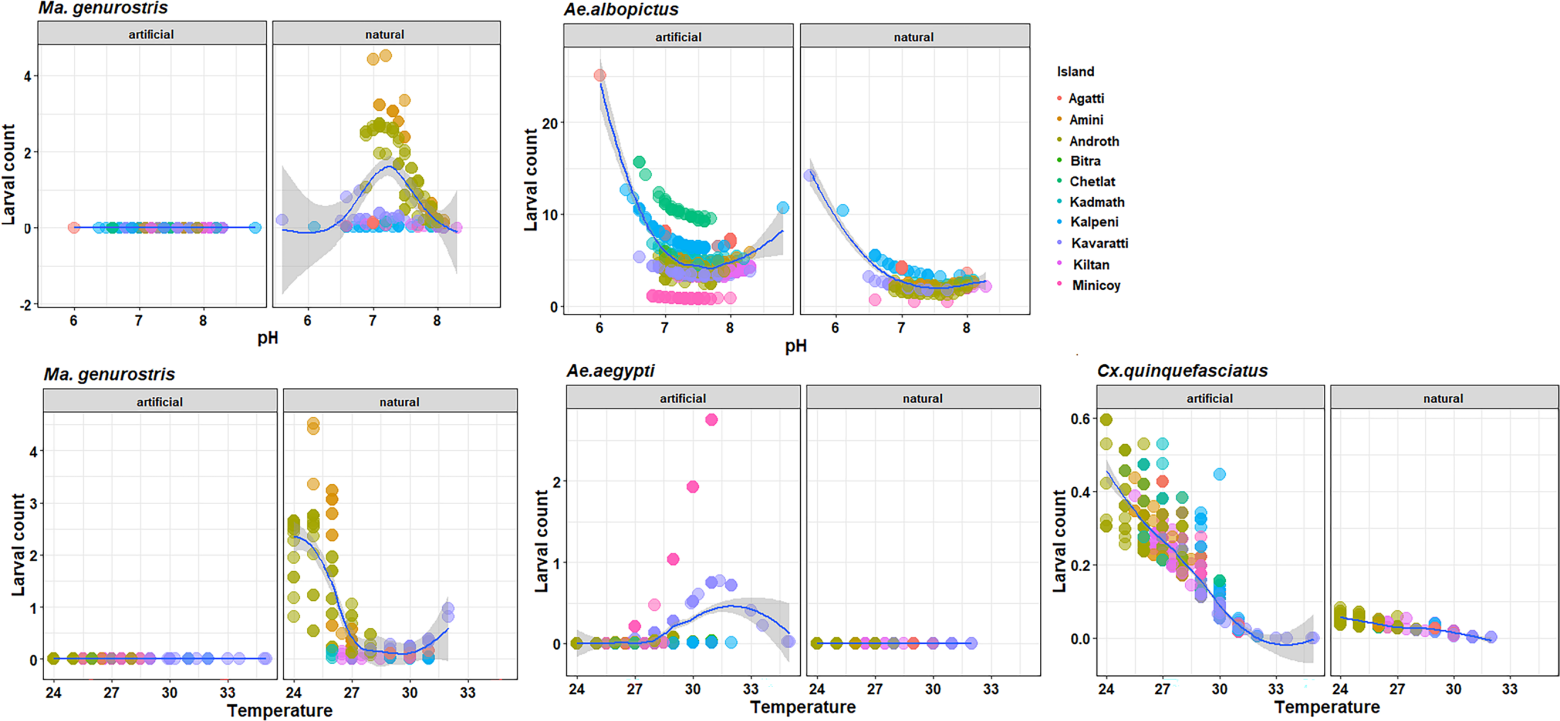
Relationship between microclimate variables and mosquito larvae abundance (count) by species and habitat type across the Lakshadweep islands.

The four most abundant mosquito species—*Ae. albopictus*, *Cx. quinquefasciatus*, *Ar. subalbatus*, *Ma. genurostris* showed variable relationship with habitat types and microclimate. The density of larval habitat positive for *Ae. albopictus* using the best fitting B-spline showed a significant negative effect of pH (χ^2^ = 9.92, df = 3, *p* < 0.01) in both natural and artificial habitats (χ^2^ = 27.78, df = 1, *p* < 0.0001; Fig. 6). However, the larval density for *Ae. albopictus* showed no significant association with temperature (χ^2^ = 5.48, df = 3, *p* = 0.13). The density of larval habitat positive for *Ae. aegypti* was marginally significant for temperature (χ^2^ = 6.67, df = 3, *p* = 0.08). Both *Ma. genurostris* and *Ar. subalbatus* were restricted to natural habitat. The density of larval habitat positive for *Ma. genurostris* showed highly significant association with pH (χ^2^ = 27.16, df = 3, *p* < 0.0001) and temperature (χ^2^ = 10.55, df = 3, *p* < 0.014)—a hump-shaped curve, peaking at 7 pH and dropping down at temperature between 28 and 30 °C (Fig. 6). The abundance of *Cx. quinquefasciatus* showed a significant negative effect of temperature (χ^2^ = 27.16, df = 3, *p* < 0.0001). In contrast, *Ar. subalbatus* and *Cx. sitiens* showed no significant association with either pH or temperature.

### Coexistence of mosquito assemblages in different habitats

We found that the coexistence of *Ae. albopictus* with other mosquito species (n = 864; *Ma. genurostris*, *Ae. vittatus*, *Ae. aegypti*, *Cx. quinquefasciatus, Cx. sitiens*) showed significant positive association (under null hypothesis OR = 1) with natural habitat type (OR = 6.47, CI = 1.42 to 2.31). Both *Ae. albopictus* with *Ar. subalbatus* resulted a positive association in natural habitat type (OR = 4.04, CI = 1.07 to 1.94), which implying evidence of co-existence at micro-habitat level without any interactions like competition or predation. In contrast, *Ae. albopictus* showed a positive association with *Ma. genurostris* in sharing natural habitat (OR = 95.60, CI = 3.45 to 5.93), however, a negative association with pH (OR = 0.15, CI =  − 3.43 to − 0.53). *Ar. subalbatus* showed a marginally significant negative association with other mosquito species in natural habitat type (OR = 0.47, CI =  − 1.52 to 0.01). *Ma. genurostris* found exclusively in natural habitats and *An. stephensi* was recorded in artificial habitat showed no significant association with pH and temperature with other mosquito species.

### Dengue epidemic threshold

**Figure 7:**
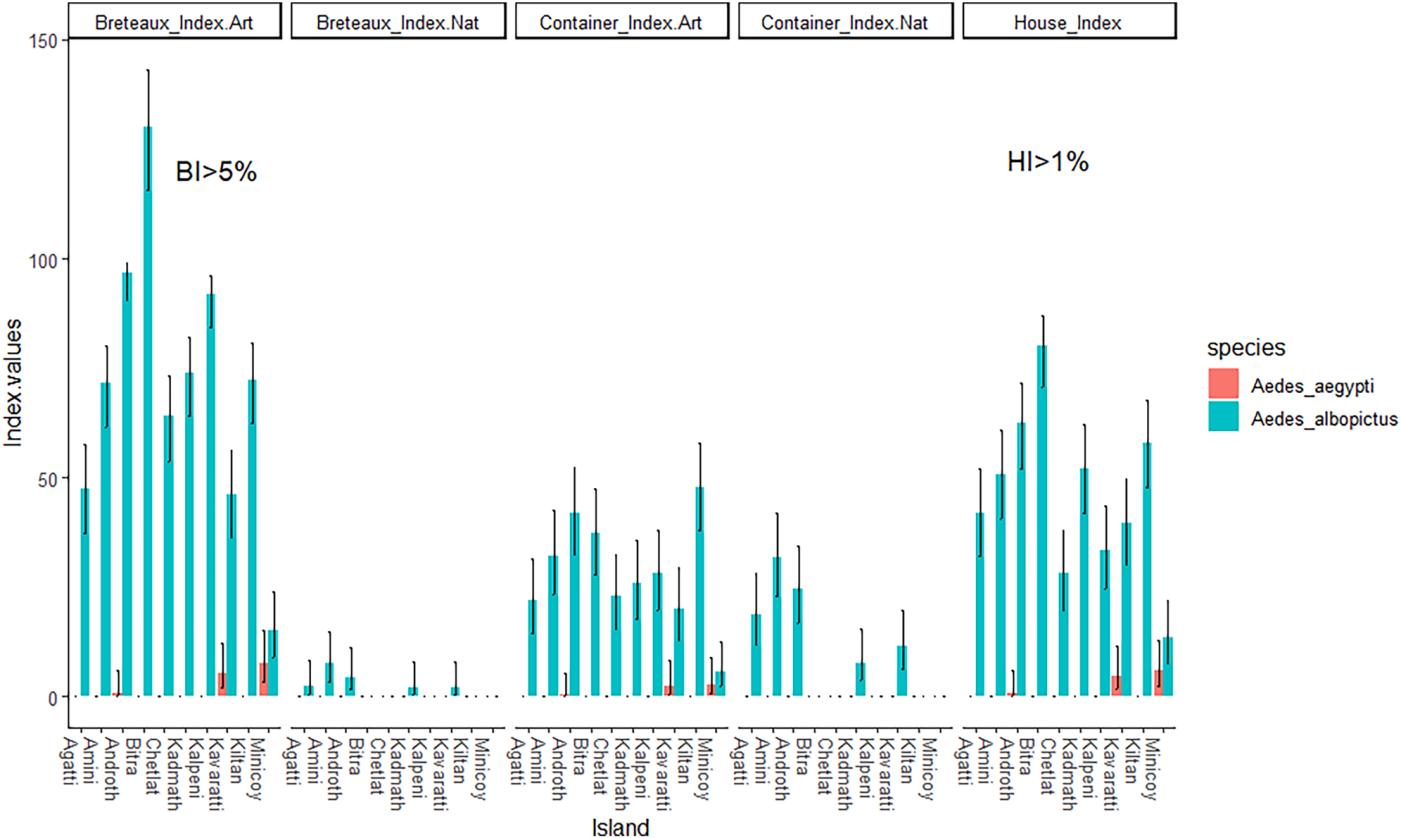
*Aedes* aegypti and *Aedes albopictus* infestation indices across islands. The error bars represent 95% confidence interval.

We calculated *Aedes* larval indices (house index, container index, and Breteau index) for each island and found that *Aedes albopictus* infestation was above the epidemic threshold across all islands with house index > 1% and Breteau index > 5% (Fig. 7).

## Discussion

Oceanic islands as natural laboratories are excellent ecosystems to understand ecological and evolutionary processes shaping the species diversity and richness. Our mosquito survey showed that fine-scale microclimatic variables drive the species abundance and co-existence patterns across a variety of natural and artificial habitats. We analyzed and identified the most productive larval habitats –discarded plastic containers and plastic drums contributing to high larval indices and above epidemic threshold across the Lakshadweep islands. Our study provides baseline information needed to devise mosquito control strategies by the removal of human-induced plastic pollution (household waste) in these remote oceanic environments which is a critical driver of disease risk.

Mosquito species diversity and distribution analysis from the Lakshadweep islands exhibited high mosquito abundance in artificial habitats. Our results revealed that mosquito community in the Lakshadweep islands is comprised of four dominant species—*Ae. albopictus*, *Cx. quinquefasciatus*, *Ar. subalbatus*, *Ma. genurostris*. Of these *Ae. albopictus*, *Cx. quinquefasciatus* are primary vectors of arboviruses and *Ar. subalbatus* incriminated as a vector of Japanese encephalitis virus^41^ and filariasis^42^ and well adapted to thrive in both natural and artificial habitats. These findings are critical for the development of a mosquito control strategy – the discarded plastic containers and plastic drums contributed to the high local abundance of larval habitat with optimal conditions and environmental resources needed for mosquito survival. It is important to consider potential sampling bias when selecting which method is most appropriate for mosquito monitoring and surveillance objectives. The combination of immature and adult mosquito surveillance methods helps detect mosquito species that are less easily detected by the adult collection methods^43^. The immature and adult collections revealed variable mosquito species diversity and richness. In larval collections, *Aedes albopictus* was the dominant species followed by *Ar. subalbatus* and *Cx. quinquefasciatus*. However, *Ar. subalbatus* was the dominant species in adult collection followed by *Cx. quinquefasciatus* and *Ae. albopictus*. In addition, we were able to estimate the expected number of species on these islands using larval collection methods whereas adult sampling remained under-sampled due to low collection frequency and variability across methods used.

We observed a positive power-law relationship between the number of species in an area and the size of an area that has been observed in other plants, animal, arthropods, pathogen communities (e.g.,^29,44^). We showed that island species richness patterns were shaped by intra-archipelago processes more strongly than by isolation from mainland source pools. We did not detect any isolation effect on the Lakshadweep islands. The z-value (slope: 2.38 ± 0.88) for mosquito richness on the Lakshadweep islands was steeper than the slope observed for other taxon on oceanic islands (0.1 to 0.4)^17^. The z-value was higher than reported for mosquito species in Melanesian archipelago (0.49) Our z-value showed a similar trend with increase in species richness, as observed in large, geographical areas or continents. This further suggest that immigration rates for mosquito are high due to ecological connectivity between the Lakshadweep islands and other environmental variables probably do not act as barriers to colonization^45^. In contrast, Melanesian archipelago is relatively far from the mainland source with low immigration rates and increased habitat heterogeneity^29^. The sampling effort was not consistent across islands using adult sampling methods which led to the lack of species-area relationship and light traps do not attract day biting mosquito species (e.g., *Ae. albopictus*). Nonetheless, our goal was to analyze and identify the most productive larval habitats and our fine scale sampling characterized the spatial ecology of mosquito species across Lakshadweep islands.

We found geographic distance between island populations was positively related to mosquito community similarity on islands. Lakshadweep islands are connected via sea routes and human transportation networks provide sufficient opportunities for island hopping, dispersal and colonisation by mosquito species. This further implies that there is no dispersal limitation which prevents ecological drift of mosquito composition across islands. Furthermore, the lower turnover rates of mosquito species within Lakshadweep islands are due to ecological connectivity between populations facilitated by urbanization and ideal environmental conditions (temperature, humidity, availability of larval habitat). . While these patterns appear to be species-specific and are primarily driven by larval ecology, ocean currents, strong winds and saltwater environment, potentially slow down the colonization process for a freshwater dwelling mosquito species. For example, we found a stark contrast in *Ae. albopictus* versus *An. stephensi*. The latter was found in very low abundance associated with freshwater habitat in eight islands. To understand colonization patterns warrants a population level genetics on the mosquito vectors to understand the extent of gene flow across islands and mainland populations.

Furthermore, larval mosquito showed a great range of ambient pH values and temperature in natural and artificial habitats. *Malaya genurostris* showed a hump-shaped curve at 7 pH (less acidic) in natural habitats. *Aedes aegypti* was found only in artificial habitats and larval abundance was zero at temperature below 28 °C and above 34 °C. *Cx. quinquefasciatus* showed a steady decline 24 °C to 33 °C. Our study showed no significant association between *Ae. albopictus* larval densities and temperature. Evans et al.^32^ and other studies showed a functional relationship between adult populations of *Ae. albopictus* and temperature. It is possible that mosquito larvae utilize different microclimates for growth where the optimum pH 7–8 plays a crucial role in maintenance of larval abundance in natural and artificial habitats.

Larval habitat sharing or spatial segregation provide insights into how species coexist and compete for limiting resources at micro-habitat scale. These findings highlight how mosquito larval abundance thrive as a function of availability of suitable environment as well as breeding sites that allows multiple species to coexist and exploit a range of micro-habitats. There are several factors like resource partitioning at microhabitat scale^46,47^, predation and cannibalism^48,49^ can be some important factors in structuring species assemblages. Our fine resolution larval habitat mapping showed a positive association between the probability of presence of larval habitat of *Ae.* albopictus and *Ma. genurostris* suggest their niches have some overlap, particularly in *Colocasia* spp. leaf axils. . However, we found a negative association at 7 pH where abundance of two species showed contrasting patterns suggesting stressful environment segregate community structure at micro-habitat level. Under laboratory conditions, *Ar. subalbatus* are voracious predators of *Ae. Albopictus*^49^. We found *Ar. subalbatus* significantly co-occurred in coconut shells (natural habitat) under the canopy partially exposed to sunlight. In general, *Ae. albopictus* showed a positive association with other mosquito species in natural habitat which was primarily driven by *Ma. genurostris* and *Ar. subalbatus*. Stage-dependent differences in size of mosquito larvae probably allows existence *Ar. subalbatus* with *Ae. albopictus* in these environments. We found a negative association between *Ar. subalbatus* and other species in natural environment. Similarly, *Cx. quinquefasciatus* positive association with *Ae. albopictus* in artificial containers with high nutrients loading of such habitats limit predator survival and reduced interspecific competition leading to high larval densities. We also found that larval density does have a negative association with larval emergence which has been one caveat in estimation of ‘true’ richness by mosquito species. Nonetheless, biodiversity indices showed that we sampled existing diversity on nearly all islands.

The *Aedes* (*Stegomyia*) indices are central to dengue epidemiological surveillance^50^. Our *Aedes albopictus* indices were estimated above the dengue epidemic threshold. While the low number of dengue cases on the islands point towards a lack of quantitative relationship between vector indices and dengue cases. Nonetheless, the high abundance of *Ae. albopictus* along with a strong association with highly prevalent non-biodegradable larval habitat suggest that there is potential for increase in arboviral pathogen transmission (e.g., dengue virus, Zika virus and chikungunya virus) in the future. Our study provides a finer resolution map of larval habitat distribution of two *Aedes* species with strikingly higher abundance of *Ae. albopictus* than *Ae. aegypti* implying asymmetrical competitive interaction between *Aedes* vectors where *Ae. aegypti* could be suppressed by *Ae. albopictus* due to its failure to outcompete at the larval stage and/or impact of interspecific mating^51–53^ which warrants a further investigation. Our finding is supported by the observed coexistence of *Ae*. *aegypti* and *Ae*. *albopictus* in similar larval habitat particularly in artificial containers. Chadee^54^ showed that pupae per person indices was better indicator at index with confirmed dengue cases compared with routine investigations. Given the sporadic arboviral cases on these islands, it is difficult to correlate larval indices and risk for dengue epidemics. The Breteaux index is considered unreliable and context dependent, nonetheless, our study design and protocols were standardized and replicated across all islands, providing invaluable information on the ecology of dengue vectors. Our study analyzed and identified the most productive larval habitats –discarded plastic containers and plastic drums contributing to high larval indices and an epidemic threshold across the Lakshadweep islands. Our data highlight the need to devise vector control strategies by removal of human-induced plastic pollution (household waste) in these remote oceanic islands which is a critical driver of disease risk.

## Methods

### Study area

The Lakshadweep archipelago [10.57° N and 72.64° E] comprises of about 36 islands of which 11 are inhabited and 16 uninhabited coral islands. The islands span over a total land area of about 32 km^2^, with a population of around 65 thousand^55^. Lakshadweep has a tropical climate with an average temperature of 27 °C–32 °C. Kavaratti is the most populated and oldest inhabited island followed by Agatti. The main occupations of the islanders include coconut cultivation, production of coir and fishing. Three ports – Kochi (Kerala), Beypore (Calicut, Kerala) and Mangalore (Karnataka)– at south-western coast of India are the major ports which connect these islands to the mainland. Lakshadweep islanders rely highly on ships and vessels for their transportation between the islands. In small islands (Bitra and Chetlat), weekly and Kavaratti and remaining islands twice a week public transport is available. There is only one airport on the Agatti island which facilitates air travel from mainland port (Kochi). Ships and vessels are available on weekly basis from mainland to all islands. There are no direct ships available for Bitra.

### Mosquito sampling and identification

We extensively surveyed ten inhabited islands (Agatti, Kavaratti, Chetlat, Kalpeni, Amini, Kiltan, Kadmath, Androth, Bitra and Minicoy) for larval habitat and species diversity and distribution during post southwest monsoon period from 1^st^ October 2019 to 13^th^ January 2020 (Fig. 1). Our main goal was to quantify the extent larval habitat use and prevalence and niche overlap between different mosquito species. Therefore, the larval sampling strategy on each island was to cover around ten percent of houses of the total population size (Table S1, Fig. 1). The houses were selected randomly on each island. We used adult collection methods to capture the existing diversity and species richness of mosquitoes on these islands. Both natural habitats such as tree holes, coconut shells, plant axils and artificial habitats such as discarded plastic containers, discarded utensils, tires, boats, grinding stones etc. were surveyed around human habitation. Mosquito larvae representing different development stages were collected using 350 mL larval dippers and maintained in mobile laboratory in cages until emergence. Each sample collection location was recorded for geographic coordinates, pH and temperature using digital meter (pH-80 HM Digital), and salinity using portable salinity refractometer (0 to 100 ppt; Extech Instruments RF20). We did not use salinity in subsequent analysis due to zero values across all habitats. We use *leaflet* package in R v.4.1.1 to map larval sites on each island.

For adult mosquito collection, we used two commonly used methods: light traps and the resting collection (Service, 1993). Mosquitoes were collected using aspirators in randomly selected houses during dawn (06:30 to 08:00) and dusk (18:30 to 21:00) for 15 min. Light traps were deployed from dusk to dawn at a height of 6 feet from the ground level near human dwelling at least once per week on each island (see Table S1). The number of traps per island varied with the size of the island. Mosquito were sorted by sexes and identified using standard morphological keys^56,57^ and stored in 80% ethanol until further analyses.

### Ethics declarations

This study was conduction with permission number F. No. LD-04001/2/20l7-S&T-UT-LKS/39 dated 16.7. 2019 from the Directorate of Science and Technology, Lakshadweep administration, Government of India.

## Data analyses

### Mosquito species diversity and richness

We investigated the relationship between larval habitat types (artificial and natural) on each island and standard community indices (such as Shannon–Wiener index and Chao1 estimator). We compared these community indices for adult collection data. Since mosquito species richness and island size could correlate with sampling effort^16,58^, we evaluated sample sufficiency by plotting sample-based species accumulation curves. The total richness was estimated by abundance-based the Chao1 estimator^59^, to calculate the cumulative mosquito richness and a 95% confidence interval (Table S2). This estimated our success in sampling the available mosquito species from all islands using the *vegan* package^60^. Kruskal–Wallis test was used to test for differences in means between habitat type and islands. All analyses were conducted in R version 3.5.2^61^.

### Species-area and species-distance relationships

We conducted two sets of analyses on mosquito richness (*S*) using larval and adult collection methods. We evaluated the species-area relationship with effects of island area (*Area*) and distance to the nearest neighbouring islands (*proximity*), the distance between islands and isolation (*Isolation*) from the mainland where we considered the distance from the three mainland ports—Kochi (9.9312° N, 76.2673° E), Calicut (11.2588° N, 75.7804° E) and Mangalore (12.9141° N, 74.8560° E) on mosquito species richness (*S*). The size of the islands was retrieved from https//lakshadweep.gov.in web source and the distance from the ports was retrieved using Google Earth ver. 7.3.4. Using general linear models (GLM) with poisson errors, eight candidate models were tested (Table 1 and Table S1):

*S* = a + b*log *Area*—semi-log species-area variant of the Arrhenius power function (*S* = CA^z^)
S = a + b*log *Isolation* (three models with ports connected to these islands)
S = a + b*log *Proximity*
S = a + b*log *Area* + c*log *Isolation*
S = a + b*log *Area* + c*log *Proximity*
6. S = a + b*log *Area* + c*log *Isolation* + d*log *Area* * log *Isolation* (three models with ports connected to these islands)
S = a + b*log *Area* + c*log *Proximity* + d*log Area* log *Proximity*
S = a (Null Model)

Where *a* was the fitted intercept and *b*, *c*, and *d* were the fitted slopes.

For all models included in the top-model set, we calculated McFadden’s pseudo-R^2^ values to estimate model fit, for which larger values suggest a better fit^62^. We used the AIC^63^ to select the best-fit model and models were ranked using small-sample-corrected AIC (AICc). Models with a difference (ΔAICc) of ≤ 4 are as parsimonious as the best-fit model (lowest AICc).

### Distance-decay relationship

To explore beta diversity patterns, we used larval mosquito abundance matrix on each island to calculate the Bray–Curtis index^64^. The rate of distance-decay of the mosquito communities was calculated as the slope of a linear least squares regression on the relationship between (ln transformed) geographic distance versus (ln transformed) mosquito similarity. Because the datapoints (pairwise comparisons) were non-independent, we used Mantel tests (r)^65,66^ with 9,999 permutations to examine the statistical significance of the distance-decay slope.

### Ecological association between larval abundance, habitat types and abiotic factors

To determine if abundance of mosquito species (with > 50 individuals) on each island differed by larval habitat type (artificial and natural) and microclimate of breeding site, we used a generalized linear mixed model (GLMM) to test the effect of habitat type and microclimate variables (pH and temperature) on the larval abundance (count) of a species and including islands as random effect (Table S3). The microclimate variables (pH and temperature) were fit using a basis-spline (B-spline) function to allow for non-linear relationships with larval density. Models used a logarithmic link function. The statistical significance of predictor variables was assessed by comparing fitted models to a null model using a likelihood ratio test. GLMMs were fit using the *glmmTMB* package. Scaled residuals of the models were inspected for overdispersion and uniformity using the *DHARMa* package^67^.

### Coexistence of mosquito assemblages in different habitats

We used a GLMM model where the presence of one species fitted was assessed as a function of presence of a second species and temperature, pH (microclimate) and habitat type (natural and artificial) with binomial distribution and islands as a random factor (Table S4). We fitted four models: (i) *Aedes albopictus* with *Armigeres subalbatus*, (ii) *Aedes albopictus* with *Malaya genurostris*, (iii) *Aedes albopictus* with other species and (iv) *Armigeres subalbatus* with other species. An odds ratio value of one indicates species are associated randomly, whereas odds ratio values of greater than one or less than one indicates a positive or negative association, respectively (*p* < 0.05, CI 95%). All analyses were carried out in *lme4* package^68^.

### Assessing the relationship between vector indices and dengue transmission

Dengue entomological surveillance has been based on vector indices threshold as an early warning tool for predicting dengue epidemic. We used three commonly used indices, namely, the House index (HI: percentage of houses infested with larvae/or pupae), the Container index (CI: percentage of water holding containers infested with larvae/or pupae), and Breteau index (BI: percentage of positive containers inspected in total houses), as quantifiable measure to determine the distribution and density of dengue mosquito populations as recommended by the WHO^69^.

## Supplementary Information

Supplementary Information 1.

Supplementary Information 2.

Supplementary Information 3.

## Data Availability

All data generated or analysed during this study are included in this published article (and its Supplementary Information files).
