# Supplementary figures and images for: Island biogeography and human practices drive ecological connectivity in mosquito species richness in the Lakshadweep Archipelago
Source: Sci Rep. 2022 May 16;12:8060. doi: 10.1038/s41598-022-11898-y (PMC9110355; doi:10.1038/s41598-022-11898-y)

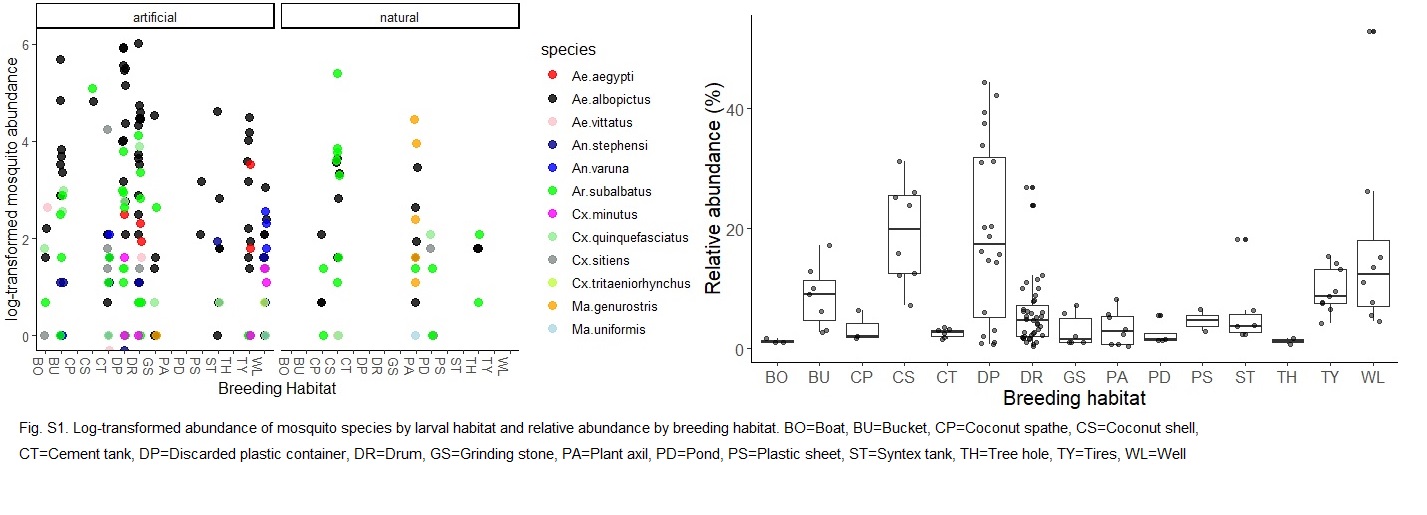

Supplement: Supplementary file 1 — Supplementary Information 1. [file 41598_2022_11898_MOESM1_ESM.jpg]

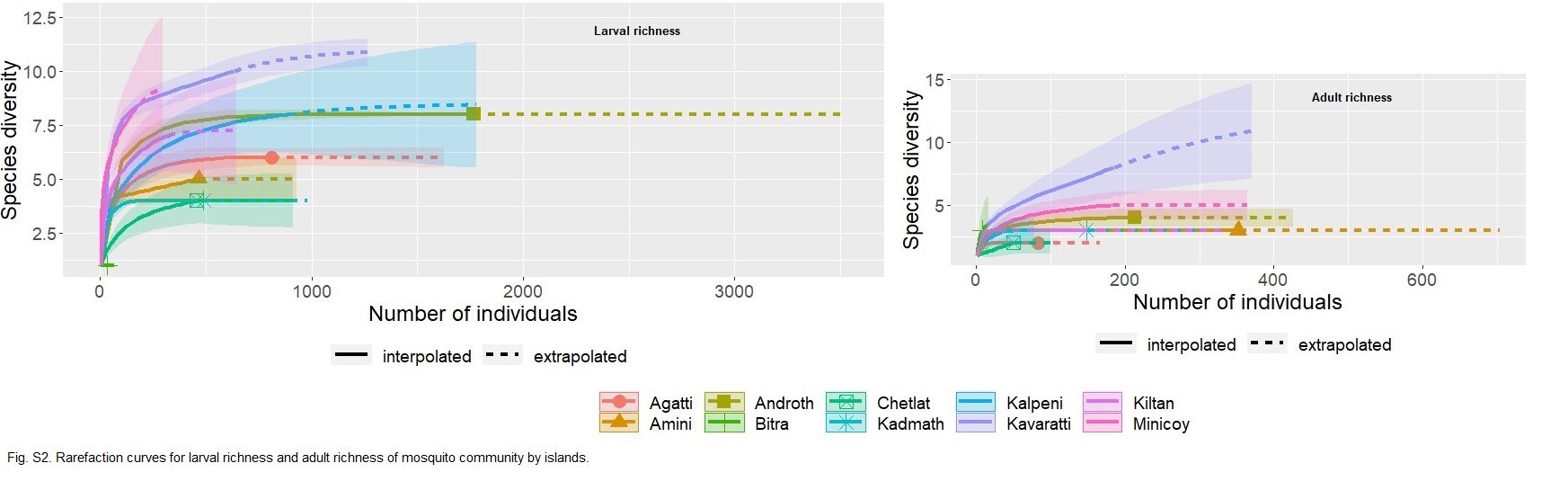

Supplement: Supplementary file 2 — Supplementary Information 2. [file 41598_2022_11898_MOESM2_ESM.jpg]
